# Aftermath

**Published:** 1896-10

**Authors:** 


### AFTERMATH.

"Our Buffalo meeting"—

The Old Guard were there, and many were the pleasant greetings to the Rayner brothers, Foelker, and Rectenwald. They are always steadfast to Association work and interests.

The Niagara Tower proved a very attractive point from which to view the ever-wonderful Falls and the beautiful country on the American and Canadian shores.

Those who were detained at home by providential and professional circumstances will only learn during the current issues of the different veterinary periodicals of the multitude of pleasures missed.

Nashville Centennial exhibition, cheap railroad rates, Southern hospitality, and our new members in the South will be strong factors in deciding the place of meeting for 1897.

It was good to see the zeal and interest in the work of our Association by such honored members of the profession as Prof. McEachran, of Montreal; Prof. Smith, of Toronto; Drs. Hinman and Thompson, of Manitoba; Elliott and Gibbs, of Ontario; and Jakeman, of Nova Scotia. The fraternal spirit of pride of progress imbues the whole profession and links together not only States, but provinces and countries.

Members Hughes, Trumbower, and Wright enjoyed every hour of the convention, and we know they will do strong missionary work among the veterinarians of Illinois in behalf of the meeting of 1897.

Ohio had a stronger representation than at any former meeting the U. S. V. M. A., and we know it will bear good fruit in 1897, no matter where the convention may be held. Meyer, Sr. and Jr., of Cincinnati, were much missed. There is good material among those who were present from which to select a strong State Secretary for the Buckeye State.

Maine and Rhode Island were the only States east of the Allegheny Mountains without a representation. This should not have been.

Among those who contributed to the pleasure and enjoyment of our sojourn at Buffalo, and who also proved a veritable guide in the convention city, was our esteemed colleague, Veterinarian Willganz.

Many new faces in the profession and Association were to be noted among those present; but it is at their homes that their influence for good is felt, for we find them earnestly at work in their State and local Associations. Among these were to be found such workers as Drs. Ellis, Pope, Jr., R. P. Lyman, Wight, Shephard, Williams, and others.

Members Ackerman, Pendry, Berns, and Bell should at once realize the necessity of re-establishing the Long Island Veterinary Medical Association. It is a good centre for strong work, and there are plenty of earnest devotees of the profession in that district to sustain the movement.

Member Liautard's cablegram and letter told forcibly where his thoughts and well-wishes were during convention hours.

The *Horseshoers' Journal*, of Chicago, for September, contained much information about the Buffalo Convention.

Messrs. Reynders & Co., of New York City, presented at the Buffalo University one of the most complete exhibitions of veterinary instruments we have ever had the pleasure of looking upon.

Messrs. Haussman and Dunn, of Chicago, had a splendid display of veterinary instruments at the Genesee Hotel.

